# A historical review of giant kelp harvesting in Tasmania

**DOI:** 10.1111/jpy.70015

**Published:** 2025-04-18

**Authors:** Hunter Forbes, Wouter Visch, Scott Bennett, J. Craig Sanderson, Jeffrey T. Wright, Cayne Layton

**Affiliations:** ^1^ Institute for Marine and Antarctic Studies University of Tasmania Hobart Australia; ^2^ Centre for Marine Socioecology University of Tasmania Hobart Australia; ^3^ Sanderson and Associates Marine Environmental Consultants Mount Nelson Tasmania Australia

**Keywords:** alginates, Australia, historical ecology, industry, macroalgae, *Macrocystis pyrifera*, management, seaweed

## Abstract

Kelps have a long history of human use and exploitation. Knowledge of past harvesting practices offers insights into environmental baselines and the contemporary management and conservation of these critically important ecosystems. In Tasmania, Australia, giant kelp (*Macrocystis pyrifera*) was commercially harvested for alginate production from 1964 to 1973, but those forests have since undergone precipitous declines due primarily to climate change. We reviewed a collection of archival data and sources to describe the history, methods, and scale of this understudied and largely forgotten industry. We calculated that >65,000 tonnes (wet weight) of *Macrocystis* were harvested from eastern Tasmania over a decade (mean annual harvest = 6531 t), making it one of the largest wild harvest industries to ever exist in the region. However, the industry had challenges finding sufficient biomass to sustain operations, ultimately driving its closure in less than a decade. Feasibility surveys prior to harvesting suggested much greater kelp availability than was ultimately realized, perhaps motivating overexpansion. Against a backdrop of climate change in this ocean warming hotspot, harvest efforts grew wider and more intensive, and during summer months when stocks were lowest, almost all exploitable biomass was harvested. It remains unclear whether harvesting contributed to the decline of Tasmanian *Macrocystis* forests, but it may have reduced their resilience and exacerbated other stressors, particularly in heavily harvested areas. This historical review provides a rare opportunity to examine the past scale and use of now‐endangered *Macrocystis* forests and also to help inform the contemporary management and conservation of seaweed resources.

**Abbreviations**
CSIRO: Commonwealth Scientific and Industrial Research Organization
SST: sea surface temperature

## INTRODUCTION

The seaweed industry is rapidly growing in scale, value, and public interest (Buschmann et al., 2017; Food and Agriculture Organization [FAO], 2024; Visch et al., 2023). Globally, the industry produced 37.8 million tonnes in 2022, of which 97% came from aquaculture (Buschmann et al., 2017; FAO, 2024). Although wild harvest contributes only a small but important proportion of current global seaweed production, humans have been utilizing seaweeds and the associated ecosystems for 1000s of years, with potentially critical anthropological and ecological consequences (e.g., nutrition, dispersal, trophic cascades; Dillehay et al., 2008; Erlandson et al., 2015; Scarborough et al., 2022).

In Tasmania, the southernmost island state of Australia, there is a deep history of seaweed use, with temperate seaweeds having played important roles in the cultures and traditions of Indigenous Australian people for at least 40,000 years, and which continues today (Hurd et al., 2023; Thurstan et al., 2018). However, wild seaweed harvest for commercial purposes only began in Tasmania 60 years ago with the establishment of the “Alginates (Australia) Company” (hereafter Alginates Australia) in 1964. Based near the town of Orford on Tasmania's east coast, the company harvested giant kelp, *Macrocystis pyrifera* (hereafter *Macrocystis*), across eastern Tasmania until its closure in 1973 (Alexander & Cassidy, 2005; Kean, 2021). *Macrocystis* was harvested for the production of alginate: a polysaccharide widely used as an emulsifier, stabilizer, and thickening agent (Hurd et al., 2014). Yet half a century later, relatively few records of this industry remain, and while it endures in some local memories (e.g., Kean, 2021), it has largely been forgotten (Hurd et al., 2023; Mac Monagail et al., 2017; Visch et al., 2023).

Alginates Australia is the only commercial harvester of *Macrocystis* to have ever operated in Australia, and it remains one of only a few Australian seaweed harvesting operations more generally. Today, Tasmania (and Australia more broadly) is home to a small but burgeoning seaweed industry, particularly focused on aquaculture but with valuable wild harvest industries for beach‐cast *Durvillaea* spp. (Fucales) and for the introduced wakame (*Undaria pinnatifida*, Laminariales; Hurd et al., 2023; Visch et al., 2023; Fisheries Research and Development Corporation [FRDC], 2024).

Understanding the historical context and commercial harvest of *Macrocystis* forests in Tasmania is also of particular importance because these ecosystems have experienced severe declines in recent times. Floating canopies of *Macrocystis* were once an iconic and ubiquitous feature of the Tasmanian coastline, with accounts of forests dense enough to limit boat passage and of individual patches almost a kilometer in diameter (Cribb, 1954). Unfortunately, since the 1960s those underwater forests have declined by 95% around Tasmania, due to climate change impacts such as ocean warming and changing oceanography (Butler et al., 2020; Johnson et al., 2011). These impacts may have also been compounded by other stressors (of partial or unknown significance), including urchin overgrazing, commercial harvesting, boat traffic, sedimentation, and poor water quality (Ling & Keane, 2024; Sanderson, 2003). In 2012, the precipitous declines also prompted the national recognition of Australia's giant kelp forest ecosystem as an “endangered ecological community” (Department of the Environment, 2022, Recover Plan Decision section; Forbes et al., 2024)—the first such listing for a marine community in Australia. The declining trend of *Macrocystis* losses in Tasmania shows the large spatial and temporal variability typical of *Macrocystis* globally (Dayton et al., 1984). Moreover, available data are mostly limited to aerial or satellite records of surface canopy cover such that many details of historical stocks and the timeline of decline remains unclear (Butler et al., 2020; Johnson et al., 2011; Sanderson, 2003). Although Alginates Australia harvested *Macrocystis* during the 1960–1970s, concurrent with the present baseline from which the dramatic losses have been assessed, it is unclear whether commercial harvesting contributed to the overall decline.

Here we have used a unique collection of unpublished archival sources to review the history, methods, and management of the *Macrocystis* harvest industry in Tasmania during the 1960s–1970s. We have reviewed the establishment, operations, and eventual closure of this understudied industry, which operated at a pivotal time of decline in Tasmanian *Macrocystis* forests. Ultimately, a better understanding of this industry offers new insights into the historical ecology and extent of this now‐endangered ecosystem, particularly given the growing focus on its conservation and restoration (Forbes et al., 2024; Layton & Johnson, 2021) and southern Australia's rapidly developing seaweed industry (Hurd et al., 2023; Visch et al., 2023).

## MATERIALS AND METHODS

We collated a variety of records to examine the history and operations of the *Macrocystis* harvesting industry in Tasmania. In particular, we reviewed a collection of previously unpublished archival sources dating from 1957 to 1987, including many hand‐ or type‐written primary sources. These comprised both qualitative and quantitative data, including harvest records, stock surveys, reports, correspondence, licenses and applications, newspaper articles, and maps. These documents discussed various harvesting companies (detailed in Results) but primarily relate to the single business that operated commercially in Tasmania: Alginates Australia. Those archival data were supplemented with a review of other published materials or secondary literature referencing the industry, of which there were few.

Archival data included records of annual *Macrocystis* harvests for Alginates Australia spanning 1964–1973, the company's entire period of operation. These data were reported in two separate documents, one for 1964–1971 in tonnes wet weight, and the other for 1970–1973 in tonnes dry weight. Using the data from 1970 to 1971 when there were parallel records of monthly harvests in both wet and dry weights, we calculated a wet‐to‐dry weight conversion factor (0.055) to estimate the corresponding weight for the times when only a single metric was reported.

Additionally, the harvest records from 1970 to 1973 were provided as monthly harvest totals. This allowed us to examine seasonal patterns in harvest activity over that period. We also paired those monthly harvest totals with historical sea surface temperature measurements from the long‐running Maria Island oceanographic station (Integrated Marine Observing System [IMOS], 2024), located on the east coast of Tasmania <25 km from the Alginates Australia facility.

Also in the archival sources were comprehensive records of harvesting trips made by Alginates Australia in 1970 and 1971, which included the length of each trip, the harvest sites, and the quantity of *Macrocystis* harvested. From these daily data, we examined seasonal and geographical patterns in harvest. Those harvest sites were grouped into eight coastal regions by the company, which it also used to report *Macrocystis* stocks (details below). We considered those sites only as approximations, given that some of the recorded names are unknown today or cover a relatively large or vague area based on the contemporary place name.

A final primary document contained records of *Macrocystis “*stocks*”* across the east coast and in each month throughout 1970 and 1971. It was unclear to what exact stock these records referred to, but given the context of the document (as information to guide harvesting and support management decisions) and that similar types of data were provided by earlier Tasmanian *Macrocystis* reports (e.g., Cribb, 1954), we presume the information pertains to the potentially harvestable biomass (i.e., the surface canopy down to ~1 m depth, rather than the total biomass). Similarly, although no details were provided to explain how Alginates Australia produced those numbers, based on descriptions and prior methods (e.g., Cribb, 1954), it seems likely they were derived from estimates of forest area multiplied by an averaged known yield per unit area. From the provided dataset, we also calculated the proportion of that potential biomass that was actually harvested each month over the 1970–1971 period.

All analyses were undertaken in R 4.2.1 (R Core Team, 2022), with figures produced using the package ggplot2 (Wickham, 2016) and maps produced using ggmap (Kahle & Wickham, 2013). All currency values provided herein are adjusted to 2024 equivalencies with the use of the Reserve Bank of Australia (RBA) inflation calculator (RBA, 2024). Similarly, all weights from the archival sources were converted from British Imperial tons to metric tonnes.

## RESULTS

### History, methods, and management of the industry

There had been interest in commercially harvesting *Macrocystis* in Tasmania since at least the early 1950s, when the Commonwealth Scientific and Industrial Research Organization (CSIRO) commissioned surveys and experimental harvests to assess *Macrocystis* stocks in eastern Tasmania (Cribb, 1954). In 1958, a license for commercial harvest of *Macrocystis* was granted to “Button, Chegwidden, and Kearns” (Alexander & Cassidy, 2005). This was the first, and only, such license ever granted in Tasmania. The licensees were in correspondence with the “Surveyor General & Secretary for Lands” in Hobart for several months prior to the granting, discussing proposed license conditions, the granting of a second license to another applicant, and a ban on harvesting during southern rock lobster (*Jasus edwardsii*) spawning season from mid‐September to mid‐December (Pownall, 1964). During that correspondence, the licensees questioned the economic feasibility of some conditions, and although it appears that the second license was never granted, the 3‐month ban on harvesting during lobster spawning season remained as a precautionary restriction until at least 1965, when a joint research program between CSIRO and Alginates Australia concluded that kelp harvesting had no effect on lobster stocks (Olsen, 1965).

**FIGURE 1:**
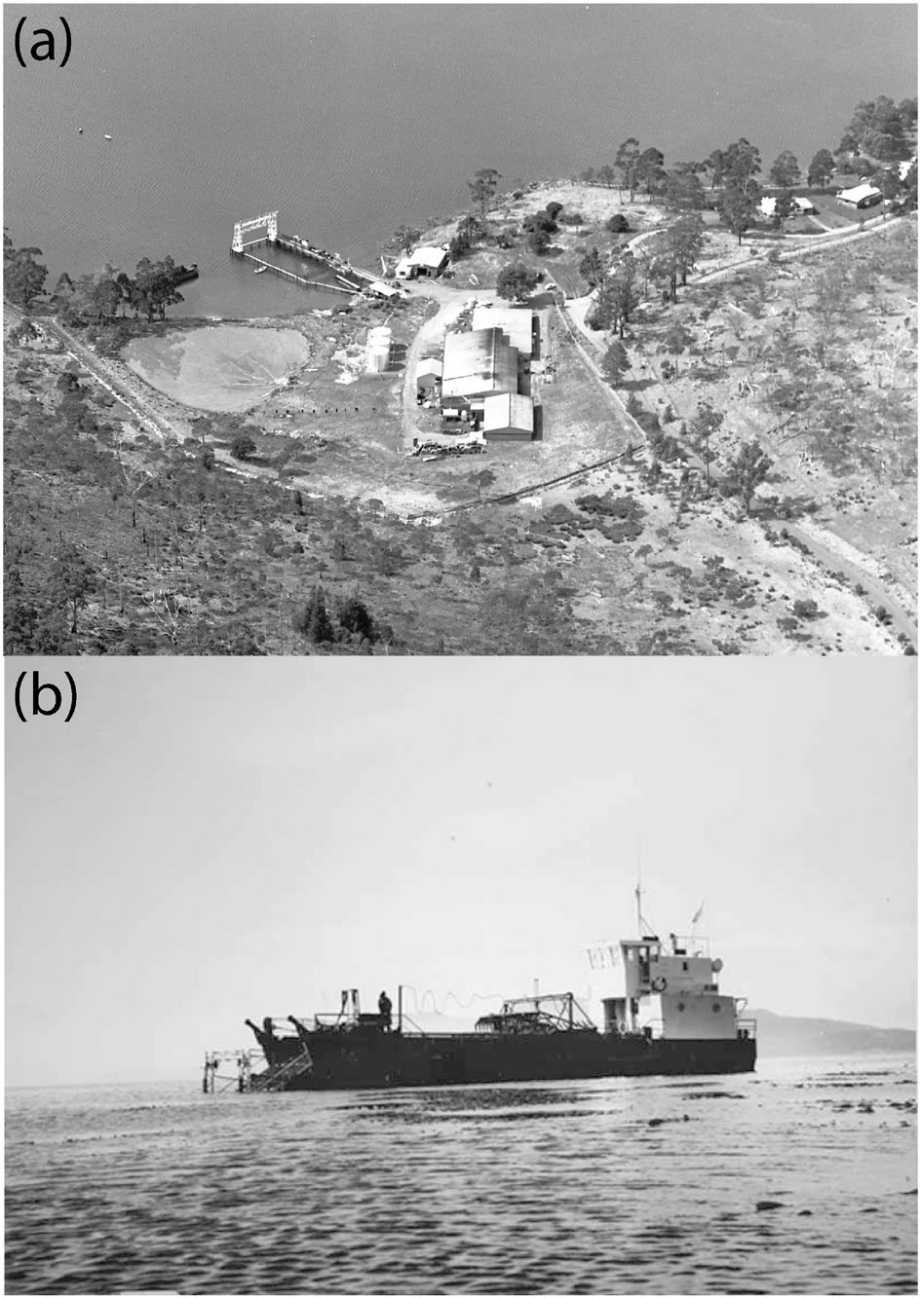
(a) Aerial view of the Alginates Australia kelp processing factory near Orford, on the east coast of Tasmania, with the docking facility and kelp holding pen visible at the upper left (Tasmanian Archives: AB713/1/12112). (b) The vessel *Alga* harvesting *Macrocystis pyrifera* near Orford (Tasmanian Archives: PH30/1/8612).

In 1961, that single extant license was transferred to Alginates Australia, a newly formed subsidiary of Marrickville Holdings, a Sydney‐based food processor (Alexander & Cassidy, 2005; Kean, 2021; Pownall, 1964). Alginates Australia explained that “*Macrocystis* weed was chosen because of the quality of alginate obtainable and the ease with which this type of weed can be mechanically harvested.” Alginates Australia established a factory near Orford, on the east coast of Tasmania (Figure 1a), and began harvesting *Macrocystis* in 1964. The cost of establishing the operation was reportedly ~25,000,000 AUD (inflation adjusted to 2024; Pownall, 1964), which seems largely due to the cost of constructing the processing factory and unloading facilities, and other capital investments (e.g., vessels). Of particular note is that the factory used >7.5 million liters of freshwater a week to process the kelp and alginate, at an annual cost of ~330,000 AUD (inflation adjusted to 2024) and which was sourced from the then‐newly built water reservoir nearby on the Prosser River (Pownall, 1964).

*Macrocystis* was harvested by cutting it ~1 m below the surface, using blades attached to a boom that extended from the front of a vessel (Figure 1b). In the beginning in 1964, the harvesting vessel *Lindy* (a converted army landing craft) and barge *Ida Bay* were used in tandem: the former harvesting kelp and the latter transporting it back to the factory under tow by the tugboat, *Maydena* (Pownall, 1964). In this way, up to 300 t of kelp could be harvested each day. It is unclear how long this arrangement was maintained, but by sometime between 1967 and 1971, only a single harvesting vessel was in use. The *Alga* was purchased in 1967 and could perform both the cutting and storage/transport roles (Figure 1b). That vessel initially had a capacity of 40 t, but was lengthened in August 1971 to increase capacity to 66 t.

Once back at the facility, the kelp was held in an ocean pen (Figure 1a) before being mechanically removed, chopped, and pumped into the factory for the alginate extraction process. At least some of the processed alginates were exported to the United Kingdom and Southeast Asia, and the domestic alginate industry at the time was valued at ~10 million AUD per year (inflation adjusted to 2024; Pownall, 1964).

Alginates Australia was required to lodge harvest returns to the Tasmanian Lands Department as a condition of it license (Sanderson, 1987, 2003). Likewise, harvesting was only allowed during daylight hours, based on concerns about the potential impact on settling lobster larvae. Harvesting was concentrated in the region immediately surrounding the factory (within ~30 km) until ~1970, after which it expanded to the entire leased area across the ~300 km coastline of eastern Tasmania from Eddystone Point in the north (41.0° S) to Fishers Point in the south (43.6° S).

In 1971, after 7 years of operation, Alginates Australia applied to expand its *Macrocystis* harvesting lease area beyond the east coast of Tasmania and also to begin harvesting southern bull kelp (*Durvillaea* spp., Fucales) as an additional source of kelp biomass, and records indicate that *Durvillaea* was processed at the facility for the first time in early 1973. We found no information regarding harvest quantities or methods of *Durvillaea*, but presumably a different method was used given its intertidal nature. The company argued that *Macrocystis* stocks across the east coast were insufficient to meet its annual harvest requirements, which had risen to ~12,000 t/year (wet weight) following the expansion of their production capacity. It had become “clear that such quantities of *Macrocystis* weed are not regularly available” and that “without access to additional supplies of seaweed, the full potential of the original project will not be realised and the plant cannot be operated profitably.”

By this time, Alginates Australia had found that east coast *Macrocystis* forests were fewer and less productive than earlier predicted and only able to reliably support total harvests of ~10,000 t/year (significantly lower than past surveys suggested, detailed below). In particular, the company found that most sites could only be harvested once annually, rather than three times as had been forecast by earlier reports. Forests were also found to be unstable and highly variable in their condition, density, and productivity among sites, seasons, and years, even in unharvested areas. Indeed, there were several instances (and always in summer) when production had to be restricted because of kelp shortages, with the company noting “decay and disappearance of weed during summer.” Also, while Alginates Australia argued that “adverse seasonal factors or environmental changes … could wipe out large areas of weed,” it reported it had “seen no evidence of overall deterioration in total weed availability during the period of factory operation.”

Altogether, it seems that while the company had noticed no significant impacts from its own harvesting efforts, it was the factory's increased production capacity and potentially the initial overestimates of harvest frequency in the earlier reports that lead to the concerns around stock availability and sufficiency. In fact, concerns about the potential scarcity of *Macrocystis* had been expressed from the outset during the initial license application in 1957: “it is quite possible that a company … could be seriously hampered by a shortage of raw materials and that this shortage could continue for periods long enough in duration to cause the company to incur heavy losses.”

Two years later at the end of 1973, Alginates Australia stopped harvesting *Macrocystis* and shut down. The last reported harvest trip was on December 5, 1973. Sources differentially attributed the closure of Alginates Australia to insufficient *Macrocystis* availability, declining condition of *Macrocystis* (including descriptions of a “rot” affecting the kelp), falling alginate prices, and/or corporate takeover (Alexander & Cassidy, 2005; Kean, 2021; Simpson, 1987). Almost all sources discussed the company's ongoing difficulties obtaining sufficient quantities of *Macrocystis*.

A decade after the closure, there was renewed interest in commercially harvesting *Macrocystis* in Tasmania and an unsuccessful attempt to resurrect the industry. In 1985, Seaweed Extracts Australia (hereafter Seaweed Extracts) applied for a *Macrocystis* harvesting license from the “Department of Sea Fisheries.” By July 1986, the initial application had progressed to a detailed business plan, with ambitions to begin harvesting in the following 6 months. Mirroring the earlier Alginates Australia lease in extent, the southern and central regions from South East Cape (43.6° S) to Maria Island (42.6° S) were to be the primary harvesting area, while the region further north from Maria Island to Eddystone Point (41° S) was designated as a backup or “reserve cutting area.” *Macrocystis* was to be harvested in the same manner as done previously (although a patented semi‐submersible vessel was also initially proposed), with an expected annual harvest of ~26,000 t (wet weight). However, unlike Alginates Australia, Seaweed Extracts also hoped to farm *Macrocystis*. The new company also intended to set up two processing plants to manufacture alginates, seaweed meal, livestock supplements, cosmetics, medical dressings, and liquid fertilizer. Ultimately though, the proposal was rejected in 1987 by the Department of Sea Fisheries, citing concerns about the sustainability and ecological impact of large‐scale *Macrocystis* harvesting, as well as doubts about the business plan and its financial viability (Simpson, 1987).

### Patterns of harvest activity

The records suggest that while Alginates Australia was operational from 1964 to 1973, it harvested a total of 65,305 t of *Macrocystis* from eastern Tasmania (wet weight; Table 1). During the 10 years of operation, the company therefore harvested an average of 6531 t (wet weight) of *Macrocystis* per year, but with high interannual variation (Table 1). The greatest annual harvest occurred in 1973, the company's final year of operation, when almost 16,000 t (wet weight) of *Macrocystis* were harvested, almost twice that of the next highest annual harvest in 1969.

**TABLE 1:** Annual harvests of *Macrocystis pyrifera* by Alginates Australia from 1964 to 1973.

Year	Total harvest (t wet weight)	Total harvest (t dry weight)
1964	533	30^a^
1965	4100	227^a^
1966	6363	352^a^
1967	8171	452^a^
1968	5935	329^a^
1969	8458	468^a^
1970	3849	212
1971	5670	349
1972	6299^a^	348
1973	15927^a^	882
Total	65,305	3649

^a^ Indicates totals that were estimated using the conversion factor between wet and dry weights (see Methods).

**FIGURE 2:**
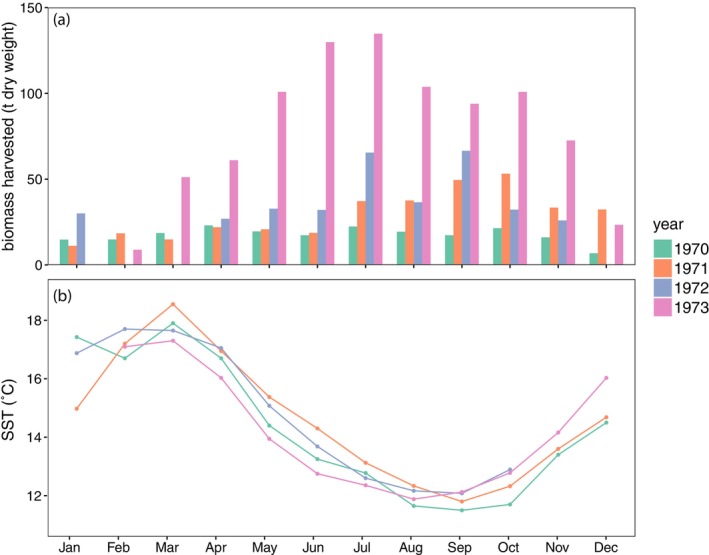
(a) Total monthly harvest (t dry weight) of *Macrocystis pyrifera* by Alginates Australia from 1970 to 1973. Note the capacity of the harvesting vessel increased in August 1971. (b) Mean monthly sea surface temperature (°C) for each year, from Maria Island on the east coast of Tasmania (IMOS, 2024).

Monthly harvest records from 1970 to 1973 showed that harvesting occurred year‐round but typically peaked in the middle of the year during the austral winter (Figure 2a). Although there was variation in harvest quantities between years and also monthly patterns, the most productive times of year were always between July and October, during late winter and early spring when water temperatures are coolest (Figure 2b). In some years, no kelp was harvested during some months of summer and early autumn (no kelp was harvested in December, February, or March of 1972 nor January of 1973). Broadly, there were no apparent differences in sea surface temperatures across all those years (Figure 2b).

**FIGURE 3:**
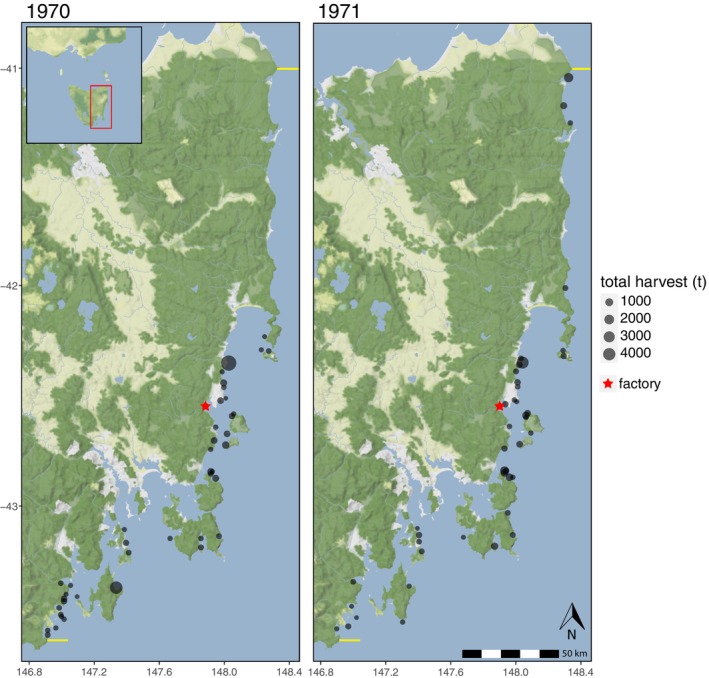
Map of *Macrocystis pyrifera* harvest sites in Tasmania during 1970 and 1971. Bubble size indicates total harvest quantity (t wet weight). The red star indicates the Alginates Australia factory at Orford. Yellow horizontal lines indicate the lease boundaries at Eddystone Point (41° S) and Fishers Point (43.6° S).

Daily site harvest records from 1970 to 1971 showed that Alginates Australia harvested *Macrocystis* along almost the entire east coast of Tasmania (and the entire lease area) during that time, covering over 300 km and 58 unique sites from “Fisher Point” in the south to “Ansons Bay” in the far north (Figure 3). Overall, its vessel made 94 harvesting trips in 1970 and 114 in 1971, with each trip lasting 1–3 days (mean = 1.4 days ± 0.6 *SD*). The vessel was filled to capacity with harvested kelp on almost every trip, and in August 1971, the vessel was modified to increase capacity from 40 to 66 t. On average, sites were visited 2.5 times a year (±3.1 *SD*); however, 23 sites (39%) were visited twice or less and 13 (22%) visited only once. Over this period, the most frequently visited site was Cape Paul Lamanon (24 visits overall; Tasman Peninsula), followed by Green Point (18 visits overall; Maria Island). Although those sites were the most frequently visited, by far the most heavily harvested site over that time was Point Bailly (total = 4719 t wet weight; Little Swanport). The second most harvested site was Adventure Bay (total = 1815 t wet weight; 15 visits in 1 year; Bruny Island), although, notably, 99% of the harvest from there was during 1970 alone. The location of one site, “Bakers Point,” could not be ascertained and thus was not included on the map (total harvest = 149 t wet weight). There were no apparent differences in seasonal harvest between regions during these years (i.e., with harvest sites allocated to regions; Figure S1).

### Stocks of *Macrocystis*

Two “extensive” surveys of Tasmanian *Macrocystis* forests were conducted prior to the establishment of the commercial harvest industry—in 1951 (by CSIRO) and in 1961 (attributed to J. Button)—each with very different results. The first was published and is well documented (see Cribb, 1954), and while we could find no record of the 1961 survey outside of the archival sources we examined, it was completed by the holding company of Alginates Australia (i.e., Marrickville Holdings and also, presumably, the same “Mr Button” who was one of the three original license holders) in cooperation with the “Fisheries Division of the Tasmanian Department of Agriculture.” Those two surveys estimated a coverage of *Macrocystis* forests in eastern Tasmania at 120 and 8 km^2^, respectively (in fact, the Cribb [1954] estimate of 120 km^2^ was only for four regions across south‐eastern Tasmania). Both surveys determined those forests had a potential yield of ~1000–1200 t/km^2^ and could sustain three harvests per year such that the estimates for annual harvests ranged from ~350,000 t (Cribb, 1954) to ~30,000 t (1961 survey by Button).

Alginates Australia documents reported a similar areal yield (mean = 1200 t/km^2^; maximum = 2000 t/km^2^) to these early surveys but found that forests could generally sustain only one harvest per year. The company also provide a broad estimate of total *Macrocystis* forest coverage along the east coast of Tasmania of 12 km^2^, giving an overall potential yield of ~6400–14,000 t/year. This is over an order of magnitude lower than the CSIRO estimate in 1951 (Cribb, 1954) but is very similar to the ~10,000 t/year that Alginates Australia determined in reality (and is likewise similar to the 1961 Button estimate, after accounting for sites being harvested only once per year).

**FIGURE 4:**
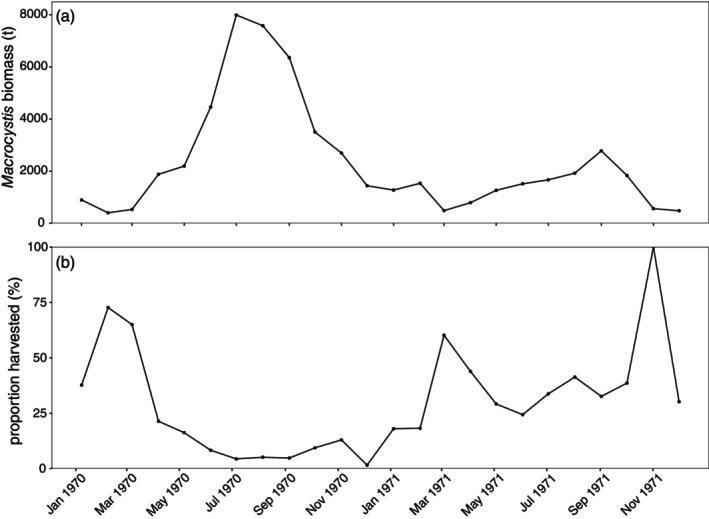
(a) Estimates from Alginates Australia of the potentially harvestable biomass (t wet weight) of *Macrocystis pyrifera* on the east coast of Tasmania during 1970–1971. (b) The proportion of the potential biomass that was ultimately harvested over the same period, based on our calculations from the reported catch data from Alginates Australia.

Alginates Australia estimated the potential harvestable biomass of *Macrocystis* across the east coast throughout 1970 and 1971 (Figure S2). Across this period, the company estimated the median harvestable biomass per month was 1602 t, but with substantial variations (Figure 4a). Peak harvestable biomass was reported in July 1970, at 7990 t of *Macrocystis*, which remained high throughout the following August and September (i.e., austral winter). Those estimates of harvestable biomass peaked during these same months in 1971, but to a lesser degree.

Alginates Australia appears to have generally harvested a substantial proportion of the available *Macrocystis* biomass during 1970–1971 (Figure 4b). Across the 2‐year period, the median proportion of the available biomass harvested was 27%. However, there was a noticeable interannual trend, with that proportion tripling from 11% in 1970 to 33% the following year. Along with this general increase, there were instances in both years when the proportion of the potential biomass that was harvested exceeded 50% (and even reached ~100% in spring 1971)—in all cases, this happened when biomass estimates were low and often soon after or before the summer (Figure 4).

## DISCUSSION

### Scale and nature of the fishery

These previously unpublished archival sources illustrate that the now largely forgotten Tasmanian *Macrocystis* industry was one of the largest seaweed wild harvesting operations to have existed in the region. The harvest of >65,000 t (wet weight) of *Macrocystis* in Tasmania was comparable to the combined harvests of all other kelps across Oceania during similar periods (Mac Monagail et al., 2017)—and has seemingly not been included in previous reporting. Even today, the Tasmanian *Macrocystis* harvest appears to be the second largest seaweed wild harvesting operation to have existed in Australia, only behind the ongoing harvest of beach‐cast *Durvillaea* in Tasmania (which has an annual yield of ~1200 t dry weight, approximately triple that of Alginates Australia; FRDC, 2024). The past harvest of *Macrocystis* even rivals many domestic animal fisheries in terms of biomass (Tuynman et al., 2023). Nonetheless, the Tasmanian *Macrocystis* fishery is outsized by the largest *Macrocystis* fisheries operating today, with Chile and Peru reporting harvests of ~20,000–30,000 t wet weight/year (Avila‐Peltroche & Villena‐Sarmiento, 2022; Nardelli et al., 2023). Even that is dwarfed by today's largest wild‐harvest kelp industry, the Chilean *Lessonia* spp. fishery (with a harvest of ~500,000 t wet weight/year; Nardelli et al., 2023). Other large *Macrocystis* fisheries have also existed historically and operated using similar methods, most notably in California (Neushul, 1989; we found no evidence that the operations of Alginates Australia were modeled on Californian *Macrocystis* harvesting or productivity). Regardless, the Tasmanian *Macrocystis* fishery remains significant, especially for its regional setting, relatively short length of harvested coastline, and trajectory of decline.

Despite its regional significance, the commercial harvest of *Macrocystis* in Tasmania was relatively short‐lived and ceased after only a decade of operation against a backdrop of apparent business constraints and changing ocean conditions. Paramount among those constraints was the difficulty sourcing sufficient quantities of *Macrocystis* to meet factory demand. The company's trip records show that by the early 1970s, the harvesters were ranging widely along the coast, traveling long distances across the entire lease area and often visiting multiple sites to fill the vessel to capacity (and presumably incurring greater vessel operating costs). This seemed particularly true in summer when standing stocks were low and kelp were affected by “rot.” Notably, the few descriptions of the rot sound similar to the necrosis and tissue loss that are regularly seen today in *Macrocystis* and other kelps in Tasmania during the summer and autumn periods of prolonged elevated seawater temperatures and thermal stress (e.g., Britton et al., 2024).

Broadly, the archival data and records illustrate a clear picture of the harvesters searching ever more widely and intensively as production demands grew and likely also as *Macrocystis* stocks declined. Conspicuously, Alginates Australia's highest annual harvest by far was in its final year of operation, suggesting even that harvest was insufficient to save the company or encourage it for the future (and was perhaps also indicative of a final intensive push to maximize the last harvest before closure). Regional climate patterns probably also influenced *Macrocystis* availability and the outlook of the harvesters, with El Niño conditions, in particular, bringing warmer sea surface temperatures and a risk of marine heatwaves—and thus reduced *Macrocystis* cover—at various times during the lifespan of the industry, including in late 1973 (Hobday et al., 2023; Wolter & Timlin, 2011). Although various factors were mentioned to have contributed to the closure of Alginates Australia at the end of 1973, it seems that *Macrocystis* unavailability was a central cause, and indeed there may have never been sufficient stock to sustain the industry's growth.

The earliest surveys of *Macrocystis* suggested that a much greater yield was possible than was ultimately revealed, suggesting that forests could sustain three harvests annually. Alginates Australia found this was not the case, and that although some small nearshore forests might be harvested two to three times annually, most large offshore forests could sustain only one harvest per year. The harvest trip data from 1970/71 indicates that sites were on average harvested 2.5 times per year, with less than a quarter of sites visited just once. This may reflect smaller partial harvests at each site or the targeting of those smaller, more productive forests, and not necessarily that harvesting was occurring beyond what was sustainable (but see discussion below).

Although the harvesting operations appear to have been largely opportunistic, the industry did face management scrutiny, including concerns from the regulator about *Macrocystis* availability and potential impacts on lobster recruitment and stock. For instance, the ban on kelp harvesting during lobster spawning season was implemented despite strong protest from the harvesters, a precautionary restriction that demonstrates consideration of the potential ecosystem‐level impacts of *Macrocystis* harvest on another fishery. However, that ban was lifted around 1965, based on conclusions that may now be more complex than previously thought (Hinojosa et al., 2015; Olsen, 1965). Alginates Australia was also required to provide spatial data on *Macrocystis* stocks in support of its lease expansion application in 1971, which might explain why higher resolution records of harvest and stocks exist for 1970/71, but seemingly not other years.

It is important to acknowledge these archival sources cannot provide an independent record of the industry, and there are likely to be gaps and details missing in what we have been able to reconstruct and the data that are available (see McClenachan et al., 2015; Thurstan et al., 2015). Unfortunately, we could find no government or otherwise “official” records of the harvests, operations, or anything related to the industry. Other information does likely exist, particularly oral histories or currently unknown personal or archival documents, which may help develop a more complete perspective of the industry (see McClenachan et al., 2015; Thurstan et al., 2015).

### Historical ecology of Tasmanian *Macrocystis* forests

The past, and even the present, extent of *Macrocystis* forests in Tasmania is uncertain, making it difficult to contextualize the scale of harvest with that of the now‐endangered ecosystem. Indeed, eastern Tasmanian waters have been warming since at least 1950, at a rate well above the global average (Johnson et al., 2011; Ridgway, 2007; Ridgway & Ling, 2023), meaning that climate stress on *Macrocystis* forests likely predates the commercial harvesting and only intensified during the industry's lifetime. Nonetheless, the records from Alginates Australia add to our knowledge of the historical extent and decline of Tasmanian *Macrocystis* forests and allow a deeper consideration of baselines and trajectories of loss (e.g., Dayton et al., 1984).

Alginates Australia estimated a *Macrocystis* forest extent of ~12 km^2^ across the east coast of Tasmania during its decade of operation (1964–1973). This is broadly consistent with the “1961 Button” estimate herein of 8 km^2^ and with contemporary analyses of aerial data that reported ~8.5 km^2^ of large floating canopies of *Macrocystis* across seven regions of the Tasmanian east coast in the early 1970s, but <1 km^2^ in the early 2000s (Johnson et al., 2011). Likewise, the most recent analysis (using Landsat satellite imagery, Butler et al., 2020) has reported a maximum canopy extent of *Macrocystis* around all of Tasmania of 4 km^2^, from 1997 to 1999, but falling to just 0.1 km^2^ by 2015. Although the early estimate of 120 km^2^ (Cribb, 1954) initially seems to have been an overestimate (Sanderson, 2003), it is possible that significant *Macrocystis* losses could have already occurred before those later estimates in the 1960s and 1970s. Indeed, given that ocean warming in the region has been evident since at least 1950 and since thermal stressors and climate‐induced losses are expected to impact a proportion of the species' range and populations (e.g., Becheler et al., 2022; King et al., 2019), it is possible that the largest absolute losses occurred in the early days when initial extents were the largest. Moreover, the recorded decline in forest extent from 120 km^2^ to 12–8 km^2^ between the two decades from the 1950s to the 1970s is proportionally similar to those declines recorded elsewhere over similar time spans (Butler et al., 2020; Johnson et al., 2011).

Historical records also help to shed light on how the structure of Tasmanian *Macrocystis* forests may have changed. Patch size, for example, seems to have changed with *Macrocystis* loss, shrinking from individual patches that reportedly reached 800 m in diameter (Cribb, 1954) to entire remnant forests today that are typically under ~400 m in diameter (Forbes et al., 2024). Forest density may have also changed, but modern estimates have recorded total biomass or plant density (e.g., Forbes et al., 2024) while the historical sources reported harvestable biomass. Overall, as *Macrocystis* forests have been lost and remnant forests modified by ongoing climate stress, there may have been a shift in forest structure from larger patches composed of more mature individuals to smaller, more transient patches dominated by younger kelp undergoing strong cycles of seasonal dieback and recovery.

### Potential ecological impacts of *Macrocystis* harvest

Given the significant declines of *Macrocystis* forests in Tasmania, there have been questions about the potential role of commercial harvesting in those losses (e.g., Kean, 2021; Sanderson, 2003). Kelp harvesting can have negative ecological impacts, both direct and indirect (e.g., Kimura & Foster, 1984; Rothman et al., 2006; Vasquez, 1995), and sustainability remains an ongoing consideration for kelp wild harvests today (Buschmann et al., 2017; Mac Monagail et al., 2017; Rebours et al., 2014).

However, assessments of *Macrocystis* harvesting outside of Australia have detected negligible impacts on forest extent or kelp growth (e.g., Borras‐Chavez et al., 2012; van Tamelen & Woodby, 2001). Although early trials from Tasmania also suggested that harvesting had limited impacts on *Macrocystis* abundance (Cribb, 1954), the nature of the mechanized commercial harvest was very different in practice, taking place over much larger areas, often more regularly, and for a longer period. Indeed, we have shown that a substantial proportion of the estimated available *Macrocystis* biomass was often harvested at any one time, particularly during summer months when stocks were lowest (Figure 4b).

The harvesting may have also had sublethal effects on *Macrocystis* fitness, further compounding other stressors and with consequences for the resilience of forests. Harvesting the uppermost portion of *Macrocystis* fronds, which includes the apical growing tip and entire surface canopy down to ~1 m depth, would have left the older and more degraded frond biomass below to senesce and die. This is consistent with how harvesters have described degraded kelp collapsing to the seafloor after harvest (herein, also Cribb, 1954; van Tamelen & Woodby, 2001). Canopy removal can also significantly reduce *Macrocystis* reproductive output (Geange, 2014; Reed, 1987) and growth, even below the cutting depth and for uncut fronds (Fox, 2016). The boat's activity itself may also have damaged the surface canopy (e.g., entanglement, propeller damage; Sanderson, 2003).

Already under stress from changing ocean conditions, the unstable and diminishing stock of *Macrocystis* in Tasmania may have been particularly vulnerable to negative effects from harvesting. Indeed, synergistic impacts from multiple stressors might have pushed *Macrocystis* populations over a tipping point, with their ability to recover further inhibited by competitive interactions with other kelps, as is being increasingly noted in contemporary observations between *Macrocystis* and the sympatric kelp *Ecklonia radiata* in Tasmania (Layton & Johnson, 2021; Ling & Keane, 2024).

In line with harvesting effort, any potential ecological impacts may have likewise been spatially heterogeneous. The records suggested harvesting was concentrated in the Mercury Passage area closest to the Alginates Australia factory—especially prior to 1970 but even after that when efforts were expanded across the full lease area. Disparate accounts have suggested that *Macrocystis* forest loss was notably severe in that region (archival records; Cribb, 1954; Sanderson, 2003; Johnson et al., 2011), and even today there remains a conspicuous gap in the species' distribution, with remnant forests only persisting to the north and south of the Mercury Passage area.

Ultimately, it is unknown, and probably unknowable, whether commercial harvesting had a widespread role in the decline of Tasmanian *Macrocystis* forests. What is clear is that harvesting can have a range of individual and population‐level impacts on *Macrocystis* and that set against a backdrop of climate change in a global ocean warming hotspot, multiple compounding impacts could have exacerbated forest decline, particularly in the most harvested areas such as the Mercury Passage.

### Lessons for the future and conclusions

The archival records have illustrated an industry that struggled with the scarcity and instability of *Macrocystis* stocks—all at a time when regional ocean temperatures were beginning to steadily increase with the then‐unrecognized onset of anthropogenic climate change. In part, these struggles also seem attributable to company overexpansion, seemingly motivated by rapid growth and apparently outdated reports of kelp availability. Growing production demands were met by ever wider and more intensive harvesting (and presumably greater vessel operating costs), which reached peaks during the summer months when *Macrocystis* stocks were lowest. Nonetheless, these demands were ultimately unable to be satisfied.

Our improved understanding of this industry now offers insights for the burgeoning seaweed industry developing in the region and for kelp conservation and management more broadly. Although commercial *Macrocystis* harvest in Tasmania collapsed, wild harvest of beach‐cast *Durvillaea* has endured (and may even have evolved from the *Macrocystis* industry), today producing ~5% of the world's alginates (FRDC, 2024; Wild Fisheries Management Branch, 2024). There were important differences between these fisheries in their harvest methods, with *Durvillaea* harvested from beach‐cast material rather than wild living forests. Nonetheless, today's *Durvillaea* fishery is managed under rules that limit licenses, harvest zones, and quantities but that lack any fishery‐independent assessments (likely due to resource constraints). Despite this, fishery‐independent data and monitoring appear essential for ensuring sustainable supply and industry development (e.g., Edgar et al., 2024). Notably, based on the industry catch data itself, the Tasmanian *Macrocystis* harvest industry did not seem to be suffering from declining harvests or stock instability. *Durvillaea* is also vulnerable to ocean warming (Thomsen et al., 2019; Visch et al., 2023), as are many other kelps of commercial interest in the region (e.g., Britton et al., 2024; James et al., 2024; Visch et al., 2023), and thus a danger of climate‐driven impacts to industry remains.

It is vital to better understand these natural resources and future‐proof these industries so that they can continue to benefit societies and economies, without collapsing themselves or compromising increasingly vulnerable ecosystems (Ward et al., 2022). Consideration of climate risk together with a precautionary principle now seems critical for successfully managing both commercial and conservation efforts in a rapidly changing marine environment (Layton, 2024; State of the Climate, 2024). The Tasmanian *Macrocystis* harvest may serve as a cautionary tale of a seaweed industry sector already lost to overexpansion, limited management, and lack of resources and knowledge in the face of climate change.

## AUTHOR CONTRIBUTIONS

**Hunter Forbes:** Conceptualization (equal); investigation (lead); writing – original draft (lead); writing – review and editing (lead). **Cayne Layton:** Conceptualization (equal); investigation (equal); writing – review and editing (equal). **Jeffrey T. Wright:** Conceptualization (equal); writing – review and editing (equal). **Wouter Visch:** Conceptualization (equal); investigation (equal); writing – review and editing (equal). **Scott Bennett:** Conceptualization (equal); writing – review and editing (equal). **J. Craig Sanderson:** Investigation (equal); writing – review and editing (equal).

## Supporting information


**Figure S1.** Mean monthly harvest (t wet weight) of Macrocystis pyrifera by Alginates Australia during 1970–1971, for geographic regions along the east coast of Tasmania (ordered from north to south).
**Figure S2.** Estimates from Alginates Australia of the potentially harvestable biomass (t wet weight) of *Macrocystis pyrifera* in regions along the east coast of Tasmania (ordered north to south) during 1970–1971.

## References

[jpy70015-bib-0001] Alexander, A. , & Cassidy, J. (2005). Kelp harvesting. *Companion to Tasmanian History*. https://www.utas.edu.au/tasmanian‐companion/biogs/E000548b.htm#:~:text=Harvesting%20began%20in%201963%2C%20with,only%20one%20crop%20per%20year.

[jpy70015-bib-0002] Avila‐Peltroche, J. , & Villena‐Sarmiento, G. (2022). Analysis of Peruvian seaweed exports during the period 1995–2020 using trade data. Botanica Marina, 65, 209–220.

[jpy70015-bib-0003] Becheler, R. , Haverbeck, D. , Clerc, C. , Montecinos, G. , Valero, M. , Mansilla, A. , & Faugeron, S. (2022). Variation in thermal tolerance of the giant kelp's gametophytes: Suitability of habitat, population quality or local adaptation? Frontiers in Marine Science, 9, 802535.

[jpy70015-bib-0004] Borras‐Chavez, R. , Edwards, M. , & Vásquez, J. A. (2012). Testing sustainable management in Northern Chile: Harvesting *Macrocystis pyrifera* (Phaeophyceae, Laminariales). A case study. Journal of Applied Phycology, 24, 1655–1665.

[jpy70015-bib-0005] Britton, D. , Layton, C. , Mundy, C. N. , Brewer, E. A. , Gaitán‐Espitia, J. D. , Beardall, J. , Raven, J. A. , & Hurd, C. L. (2024). Cool‐edge populations of the kelp *Ecklonia radiata* under global ocean change scenarios: Strong sensitivity to ocean warming but little effect of ocean acidification. Proceedings of the Royal Society B: Biological Sciences, 291(2015), 20232253. 10.1098/rspb.2023.2253 PMC10791590 38228502

[jpy70015-bib-0006] Buschmann, A. H. , Camus, C. , Infante, J. , Neori, A. , Israel, Á. , Hernández‐González, M. C. , Pereda, S. V. , Gomez‐Pinchetti, J. L. , Golberg, A. , Tadmor‐Shalev, N. , & Critchley, A. T. (2017). Seaweed production: Overview of the global state of exploitation, farming and emerging research activity. European Journal of Phycology, 52, 391–406.

[jpy70015-bib-0007] Butler, C. L. , Lucieer, V. L. , Wotherspoon, S. J. , & Johnson, C. R. (2020). Multi‐decadal decline in cover of giant kelp *Macrocystis pyrifera* at the southern limit of its Australian range. Marine Ecology Progress Series, 653, 1–18.

[jpy70015-bib-0008] Cribb, A. B. (1954). *Macrocystis pyrifera* (L.) Ag. Community and Species Profile and Threats In Tasmanian waters. Marine and Freshwater Research, 5, 1–34.

[jpy70015-bib-0009] Dayton, P. K. , Currie, V. , Gerrodette, T. , Keller, B. D. , Rosenthal, R. , & Tresca, D. V. (1984). Patch dynamics and stability of some California kelp communities. Ecological Monographs, 54, 254–289.

[jpy70015-bib-0010] Department of the Environment . (2022). Giant Kelp Marine Forests of South East Australia in Community and Species Profile and Threats Database. Department of the Environment, Canberra. Retrieved date from July 17, 2024. http://www.environment.gov.au/sprat

[jpy70015-bib-0011] Dillehay, T. D. , Ramírez, C. , Pino, M. , Collins, M. B. , Rossen, J. , & Pino‐Navarro, J. D. (2008). Monte Verde: Seaweed, food, medicine, and the peopling of South America. Science, 320, 784–786. 18467586 10.1126/science.1156533

[jpy70015-bib-0012] Edgar, G. J. , Bates, A. E. , Krueck, N. C. , Baker, S. C. , Stuart‐Smith, R. D. , & Brown, C. J. (2024). Stock assessment models overstate sustainability of the world's fisheries. Science, 385, 860–865. 39172840 10.1126/science.adl6282

[jpy70015-bib-0013] Erlandson, J. M. , Braje, T. J. , Gill, K. M. , & Graham, M. H. (2015). Ecology of the kelp highway: Did marine resources facilitate human dispersal from Northeast Asia to the Americas? The Journal of Island and Coastal Archaeology, 10, 392–411.

[jpy70015-bib-0017] Fisheries Research and Development Corporation . (2024). Seaweed fishery . https://www.fishfiles.com.au/fisheries‐and‐farms/tas‐fisheries‐and‐farms/seaweed‐fishery

[jpy70015-bib-0014] Food and Agricultural Organization of the United Nations . (2024). The State of World Fisheries and Aquaculture 2024 – Blue Transformation in action. Food and Agriculture Organization of the United Nations. 10.4060/cd0683en

[jpy70015-bib-0015] Forbes, H. , Strain, E. M. A. , Bennett, S. , Ling, S. D. , & Layton, C. (2024). Endangered giant kelp forests support similar fish and macroinvertebrate communities to sympatric stipitate kelp forests. Biodiversity and Conservation, 33, 2503–2525.

[jpy70015-bib-0016] Fox, M. (2016). Biomass loss reduces growth and resource translocation in giant kelp *Macrocystis pyrifera* . Marine Ecology Progress Series, 562, 65–77.

[jpy70015-bib-0018] Geange, S. W. (2014). Growth and reproductive consequences of photosynthetic tissue loss in the surface canopies of *Macrocystis pyrifera* (L.) C. Agardh. Journal of Experimental Marine Biology and Ecology, 453, 70–75.

[jpy70015-bib-0019] Hinojosa, I. A. , Green, B. S. , Gardner, C. , & Jeffs, A. (2015). Settlement and early survival of southern rock lobster, *Jasus edwardsii*, under climate‐driven decline of kelp habitats. ICES Journal of Marine Science, 72, i59–i68.

[jpy70015-bib-0020] Hobday, A. J. , Burrows, M. T. , Filbee‐Dexter, K. , Holbrook, N. J. , Sen Gupta, A. , Smale, D. A. , Smith, K. E. , Thomsen, M. S. , & Wernberg, T. (2023). With the arrival of El Niño, prepare for stronger marine heatwaves. Nature, 621, 38–41. 37673984 10.1038/d41586-023-02730-2

[jpy70015-bib-0022] Hurd, C. L. , Wright, J. T. , Layton, C. , Strain, E. M. A. , Britton, D. , Visch, W. , Barrett, N. , Bennett, S. , Chang, K. J. L. , Edgar, G. , Fitton, J. H. , Greeno, D. , Jameson, I. , Johnson, C. R. , Karpiniec, S. S. , Kraft, G. T. , Ling, S. D. , Macleod, C. M. , Paine, E. R. , … Willis, A. (2023). From Tasmania to the world: Long and strong traditions in seaweed use, research, and development. Botanica Marina, 66, 1–36.

[jpy70015-bib-0021] Hurd, C. , Harrison, P. , Bischof, K. , & Lobban, C. (2014). Seaweed ecology and physiology. Cambridge University Press.

[jpy70015-bib-0023] Integrated Marine Observing System . (2024). ANMN National Reference Stations – Combined long‐term hydrological data product (1944–2014). IMOS, CSIRO Oceans & Atmosphere. https://catalogue‐imos.aodn.org.au/geonetwork/srv/eng/catalog.search#/metadata/d3feff71‐ebe1‐4b66‐91c6‐149beceef205

[jpy70015-bib-0024] James, C. , Layton, C. , Hurd, C. L. , & Britton, D. (2024). The endemic kelp *Lessonia corrugata* is being pushed above its thermal limits in an ocean warming hotspot. Journal of Phycology, 60, 503–516. 38426571 10.1111/jpy.13434

[jpy70015-bib-0025] Johnson, C. R. , Banks, S. C. , Barrett, N. S. , Cazassus, F. , Dunstan, P. K. , Edgar, G. J. , Frusher, S. D. , Gardner, C. , Haddon, M. , Helidoniotis, F. , Hill, K. L. , Holbrook, N. J. , Hosie, G. W. , Last, P. R. , Ling, S. D. , Melbourne‐Thomas, J. , Miller, K. , Pecl, G. T. , Richardson, A. J. , … Taw, N. (2011). Climate change cascades: Shifts in oceanography, species' ranges and subtidal marine community dynamics in eastern Tasmania. Journal of Experimental Marine Biology and Ecology, 400, 17–32.

[jpy70015-bib-0026] Kahle, D. , & Wickham, H. (2013). Ggmap: Spatial visualization with ggplot2. The R Journal, 5, 144.

[jpy70015-bib-0027] Kean, Z. (2021, February 26). Remembering Tasmania’s underwater forests. Austrialian Broadcasting Corporation News. https://www.abc.net.au/news/science/2021‐02‐27/tasmania‐giant‐kelp‐forests‐disappearing‐global‐ocean‐warming/11209188

[jpy70015-bib-0028] Kimura, R. S. , & Foster, M. S. (1984). The effects of harvesting *Macrocystis pyrifera* on the algal assemblage in a giant kelp forest. Hydrobiologia, 116, 425–428.

[jpy70015-bib-0029] King, N. G. , McKeown, N. J. , Smale, D. A. , Wilcockson, D. C. , Hoelters, L. , Groves, E. A. , Stamp, T. , & Moore, P. J. (2019). Evidence for different thermal ecotypes in range centre and trailing edge kelp populations. Journal of Experimental Marine Biology and Ecology, 514–515, 10–17.

[jpy70015-bib-0030] Layton, C. (2024). Coasts and marine: Kelp. In Tasmanian 2024 state of the environment report (pp. 17–27). Tasmanian Planning Commission. https://www.planning.tas.gov.au/__data/assets/pdf_file/0009/782604/SOE‐Report‐2024‐Vol.2.1_27‐September‐2024.pdf

[jpy70015-bib-0031] Layton, C. , & Johnson, C. (2021). Assessing the feasibility of restoring giant kelp forests in Tasmania . Report to the National Environmental Science Program, Marine Biodiversity Hub. Institute for Marine and Antarctic Studies, University of Tasmania. https://www.nespmarine.edu.au/system/files/Layton%20et%20al_E7_M5_Assessing%20the%20feasibility%20of%20restoring%20giant%20kelp%20forests%20in%20Tas.pdf

[jpy70015-bib-0032] Ling, S. D. , & Keane, J. P. (2024). Climate‐driven invasion and incipient warnings of kelp ecosystem collapse. Nature Communications, 15, 400. 10.1038/s41467-023-44543-x PMC10776680 38195631

[jpy70015-bib-0033] Mac Monagail, M. , Cornish, L. , Morrison, L. , Araújo, R. , & Critchley, A. T. (2017). Sustainable harvesting of wild seaweed resources. European Journal of Phycology, 52, 371–390.

[jpy70015-bib-0034] McClenachan, L. , Cooper, A. B. , McKenzie, M. G. , & Drew, J. A. (2015). The importance of surprising results and best practices in historical ecology. Bioscience, 65, 932–939.

[jpy70015-bib-0035] Nardelli, A. E. , Visch, W. , Wright, J. T. , & Hurd, C. L. (2023). Concise review of genus *Lessonia* Bory. Journal of Applied Phycology, 35, 1485–1498.

[jpy70015-bib-0036] Neushul, P. (1989). Seaweed for war: California's World War I kelp industry. Technology and Culture, 30, 561–583.

[jpy70015-bib-0037] Olsen, A. M. (1965). An investigation into the effect that the harvesting of *Macrocystis* might have on the stocks of the spiny lobster or marine crayfish, *Jasus* *lalandei* . Alginates (Australia) Company and CSIRO (Fisheries and Oceanography).

[jpy70015-bib-0038] Pownall, P. (1964). Harvesting brown kelp: New industry for Tasmania. Fisheries Newsletter, 23, 11–15.

[jpy70015-bib-0064] R Core Team . (2022). R: A language and environment for statistical computing. R Foundation for Statistical Computing. https://www.R‐project.org/

[jpy70015-bib-0040] Rebours, C. , Marinho‐Soriano, E. , Zertuche‐González, J. A. , Hayashi, L. , Vásquez, J. A. , Kradolfer, P. , Soriano, G. , Ugarte, R. , Abreu, M. H. , Bay‐Larsen, I. , Hovelsrud, G. , Rødven, R. , & Robledo, D. (2014). Seaweeds: An opportunity for wealth and sustainable livelihood for coastal communities. Journal of Applied Phycology, 26, 1939–1951. 25346571 10.1007/s10811-014-0304-8 PMC4200322

[jpy70015-bib-0041] Reed, D. C. (1987). Factors affecting the production of sporophylls in the giant kelp *Macrocystis pyrifera* (L.) C.Ag. Journal of Experimental Marine Biology and Ecology, 113, 61–69.

[jpy70015-bib-0039] Reserve Bank of Australia . (2024). Inflation Calculator. The Reserve Bank of Australia. https://www.rba.gov.au/calculator/

[jpy70015-bib-0042] Ridgway, K. R. (2007). Long‐term trend and decadal variability of the southward penetration of the east Australian current. Geophysical Research Letters, 34, 13.

[jpy70015-bib-0043] Ridgway, K. R. , & Ling, S. D. (2023). Three decades of variability and warming of nearshore waters around Tasmania. Progress in Oceanography, 215, 103046.

[jpy70015-bib-0044] Rothman, M. D. , Anderson, R. J. , & Smit, A. J. (2006). The effects of harvesting of the South African kelp (*Ecklonia maxima*) on kelp population structure, growth rate and recruitment. Journal of Applied Phycology, 18, 335–341.

[jpy70015-bib-0045] Sanderson, J. C. (1987). *A survey of the* Macrocystis pyrifera *(L.) C. Agardh stocks on the East Coast of Tasmania* (Technical Report No. 21). Tasmanian Deptartment of Sea Fisheries.

[jpy70015-bib-0046] Sanderson, J. C. (2003). Restoration of String Kelp (*Macrocystis pyrifera*) habitat on Tasmania's east and south coasts. https://data.imas.utas.edu.au/attachments/9d3e6280‐44ab‐11dc‐8cd0‐00188b4c0af8/FinalTotalMacReport2003.pdf

[jpy70015-bib-0047] Scarborough, C. , Welch, Z. S. , Wilson, J. , Gleason, M. G. , Saccomanno, V. R. , & Halpern, B. S. (2022). The historical ecology of coastal California. Ocean and Coastal Management, 230, 106352.

[jpy70015-bib-0048] Simpson, S. (1987, June 7). Seaweed entrepreneur angered by knockback. *Sunday Tasmanian* .

[jpy70015-bib-0049] State of the Climate . (2024). CSIRO and Bureau of Meteorology, © Government of Australia.

[jpy70015-bib-0050] Tasmanian Archives Education Department; Teaching Aids Centre ‐ Photographic Prints – Sequence [1–12387]; AB713/1/12112. https://libraries.tas.gov.au/Digital/AB713‐1‐12112/AB713‐1‐12112

[jpy70015-bib-0051] Tasmanian Archives Unidentified Creating Agency; Miscellaneous Collection of Photographs; PH30/1/8612. https://libraries.tas.gov.au/Digital/PH30‐1‐8612/PH30‐1‐8612

[jpy70015-bib-0053] Thomsen, M. S. , Mondardini, L. , Alestra, T. , Gerrity, S. , Tait, L. , South, P. M. , Lilley, S. A. , & Schiel, D. R. (2019). Local extinction of bull kelp (*Durvillaea* spp.) due to a marine heatwave. Frontiers in Marine Science, 6, 84.

[jpy70015-bib-0054] Thurstan, R. H. , Brittain, Z. , Jones, D. S. , Cameron, E. , Dearnaley, J. , & Bellgrove, A. (2018). Aboriginal uses of seaweeds in temperate Australia: An archival assessment. Journal of Applied Phycology, 30, 1821–1832.

[jpy70015-bib-0055] Thurstan, R. H. , McClenachan, L. , Crowder, L. B. , Drew, J. A. , Kittinger, J. N. , Levin, P. S. , Roberts, C. M. , & Pandolfi, J. M. (2015). Filling historical data gaps to foster solutions in marine conservation. Ocean and Coastal Management, 115, 31–40. 10.1016/j.ocecoaman.2015.04.019

[jpy70015-bib-0056] Tuynman, H. , Cao, A. C. , Dylewski, M. , & Curtotti, R. (2023). Australian fisheries and aquaculture statistics 2022 . https://daff.ent.sirsidynix.net.au/client/en_AU/search/asset/1035343/0

[jpy70015-bib-0057] van Tamelen, P. G. , & Woodby, D. (2001). Macrocystis biomass, quality, and harvesting effects in relation to the herring spawn‐on‐kelp fishery in Alaska. Alaska Fisheries Research Bulletin, 8, 118–131.

[jpy70015-bib-0058] Vasquez, J. A. (1995). Ecological effects of Brown seaweed harvesting. Botanica Marina, 38, 251–258.

[jpy70015-bib-0059] Visch, W. , Layton, C. , Hurd, C. L. , Macleod, C. , & Wright, J. T. (2023). A strategic review and research roadmap for offshore seaweed aquaculture—A case study from southern Australia. Reviews in Aquaculture, 15, 1467–1479.

[jpy70015-bib-0060] Ward, D. , Melbourne‐Thomas, J. , Pecl, G. T. , Evans, K. , Green, M. , McCormack, P. C. , Novaglio, C. , Trebilco, R. , Bax, N. , Brasier, M. J. , Cavan, E. L. , Edgar, G. , Hunt, H. L. , Jansen, J. , Jones, R. , Lea, M. A. , Makomere, R. , Mull, C. , Semmens, J. M. , … Layton, C. (2022). Safeguarding marine life: Conservation of biodiversity and ecosystems. Reviews in Fish Biology and Fisheries, 32(1), 65–100. 10.1007/s11160-022-09700-3 35280238 PMC8900478

[jpy70015-bib-0061] Wickham, H. (2016). ggplot2: Elegant graphics for data analysis. Springer‐Verlag.

[jpy70015-bib-0062] Wild Fisheries Management Branch . (2024). Operational guide for the beach‐cast Marine Plant Fishery (2024‐25 Ed). Department of Natural Resources and Environment Tasmania.

[jpy70015-bib-0063] Wolter, K. , & Timlin, M. S. (2011). El Niño/southern oscillation behaviour since 1871 as diagnosed in an extended multivariate ENSO index (MEI.Ext). International Journal of Climatology, 31, 1074–1087.

