# Supplementary material for: A historical review of giant kelp harvesting in Tasmania
Source: J Phycol. 2025 Apr 18;61(3):574–86. doi: 10.1111/jpy.70015 (PMC12168100; doi:10.1111/jpy.70015)
Supplement: Supplementary file 1 — Figure S1. Mean monthly harvest (t wet weight) of Macrocystis pyrifera by Alginates Australia during 1970–1971, for geographic regions along the east coast of Tasmania (ordered from north to south). Figure S2. Estimates from Alginates Australia of the potentially harvestable biomass (t wet weight) of Macrocystis pyrifera in regions along the east coast of Tasmania (ordered north to south) during 1970–1971. [file JPY-61-574-s001.docx]

**SUPPLEMENTARY INFORMATION:**

**A historical review of giant kelp harvesting in Tasmania**

Hunter Forbes^1*^, Wouter Visch^1^, Scott Bennett^1,2^, J. Craig Sanderson^3^, Jeffrey T. Wright^1^, Cayne Layton^1,2^

*^1^Institute for Marine and Antarctic Studies, University of Tasmania, Hobart, Australia*

*^2^Centre for Marine Socioecology, University of Tasmania, Hobart, Australia*

*^3^Sanderson and Associates Marine Environmental Consultants, Mount Nelson, Tasmania*

* [hunter.forbes@utas.edu.au](mailto:hunter.forbes@utas.edu.au)

**Figure S1:** Mean monthly harvest (t wet weight) of Macrocystis pyrifera by Alginates Australia during 1970-1971, for geographic regions along the east coast of Tasmania (ordered from north to south).

**Figure S2:** Estimates from Alginates Australia of the potentially harvestable biomass (t wet weight) of *Macrocystis pyrifera* in regions along the east coast of Tasmania (ordered north to south) during 1970–1971.
